# A Cluster of Neuroinvasive Adenovirus Infections on a College Campus: Case Series

**DOI:** 10.5811/cpcem.1406

**Published:** 2023-05-27

**Authors:** Nicholas Valentini, Madison Breeden, Laura E. Felley, Nathan B. Roberts, Maia Dinsmore, Aaron Krumheuer, Colleen Grassley, Sacha Montas, Benjamin Bassin, Kylee Phillips

**Affiliations:** * University of Michigan, Department of Emergency Medicine, Ann Arbor, Michigan; † University of Michigan, Department of Internal Medicine, Division of Infectious Disease, Ann Arbor, Michigan

**Keywords:** adenovirus, neuroinvasive, case report, encephalitis

## Abstract

**Introduction:**

We present six adenovirus cases that emerged from a cluster of respiratory illnesses within a college population. Two patients required intensive care with complicated hospital courses and experienced residual symptoms. Four additional patients were evaluated in the emergency department (ED) with two additional diagnoses of neuroinvasive disease. These cases represent the first known occurrences of neuroinvasive adenovirus infections in healthy adults.

**Case Series:**

An individual presented to the ED with fever, altered mental status, and seizures after being found unresponsive in his apartment. His presentation was concerning for significant central nervous system pathology. Shortly after his arrival, a second individual presented with similar symptoms. Both required intubation and admission to a critical care setting. Over a 24-hour period, four additional individuals presented to the ED with moderate severity symptoms. All six individuals tested positive for adenovirus in their respiratory secretions. A provisional diagnosis of neuroinvasive adenovirus was made after consultation with infectious diseases.

**Conclusion:**

This cluster of cases appears to represent the first known reported diagnosis of neuroinvasive adenovirus in healthy young individuals. Our cases were also unique in demonstrating a significant spectrum of disease severity. Over 80 individuals in the broader college community ultimately tested positive for adenovirus in respiratory samples. As respiratory viruses continue to challenge our healthcare systems, new spectrums of disease are being discovered. We believe clinicians should be aware of the potential severity of neuroinvasive adenovirus disease.

## INTRODUCTION

We present a spectrum of neuroinvasive adenovirus cases that emerged from a cluster of viral respiratory illnesses within a college population. These cases appear to represent the first known occurrences of neuroinvasive adenovirus infections in healthy adults without immunocompromise. Clinicians should be aware of the potential for novel outbreaks of disease among concentrated populations such as those on a college campus. In addition, clinicians should be aware of the risk of atypical presentations of adenovirus infection.

## CASE SERIES

Patient one was a 23-year-old male with a past medical history (PMH) of nephrolithiasis who presented to the emergency department (ED) with altered mental status (AMS) in the setting of one week of infectious symptoms including fever, body aches, chills, headache, and cough. On arrival, the patient was actively seizing. Exam was further notable for hyperreflexia and a petechial rash on the head and upper torso. Signs of trauma were absent. The patient was intubated for airway protection and was empirically treated with dexamethasone, meropenem (due to reported allergies), vancomycin, and acyclovir. Lumbar puncture (LP) was completed with a negative Gram stain and a lymphocytic predominance. Computed tomography (CT) showed no acute pathology.

An electroencephalogram (EEG) was obtained and did not suggest ongoing seizure activity. Blood work showed a mild leukocytosis without additional acute findings. Creatinine kinase (CK) testing was normal. Serum and urine toxicologic studies were negative. A respiratory polymerase chain reaction (PCR) sample was positive for adenovirus. The patient was admitted to the intensive care unit. He underwent expanded testing for immunocompromising and infectious conditions without pertinent findings. Cerebrospinal fluid (CSF) samples were tested for adenovirus and resulted without positive PCR findings. Respiratory samples were sent for expanded genomic testing to the US Centers for Disease Control and Prevention. He was treated with cidofovir. The patient was extubated on day four of his hospital course. His mental status slowly improved after extubation; however, he had persistent deficits in executive functioning and short-term memory. He was discharged on hospital day seven, and at outpatient follow-up two weeks after discharge he continued to have residual deficits in memory and cognition.

Patient two was a 21-year-old male with an unremarkable PMH who presented to the ED due to AMS and seizure in the setting of one week of infectious symptoms including fevers, cough, and congestion. The patient was not following commands but moving all extremities without focal neurological deficits. He had no rash, although there had been reports of rash earlier in the week. The patient was intubated and empirically treated with dexamethasone, cefepime, vancomycin, and acyclovir. An LP showed a lymphocytic predominance and a negative Gram stain. A respiratory PCR was positive for adenovirus. A head CT was initially concerning for a small intracranial hemorrhage in the left parietal lobe, but follow-up magnetic resonance imaging ultimately showed this to be a cavernoma. An EEG did not suggest seizure activity. Toxicologic studies were negative. He was treated with cidofovir. He was extubated on day three of his hospital course, and his mental status slowly improved after extubation. He was discharged home at his mental status baseline after five days in the hospital. Viral cerebrospinal fluid studies including for adenovirus were negative.

Patients three and four presented 24 hours after the initial cases. They were an 18-year-old male and a 23-yearold male, respectively, with no PMH. Presenting symptoms included sore throat, fever, myalgias, cough, and neck pain. Both patients had had close contact with patient one. Physical exam in both cases was notable for nuchal rigidity, no focal neurologic deficits, and no rash or other skin lesions. Blood work was without pertinent findings. A respiratory PCR returned positive for adenovirus in both cases. An LP was performed for both individuals, again notable for a lymphocytic predominance. Both patients became febrile during their ED evaluations. Due to concern for worsening central nervous system (CNS) symptoms of headache and neck stiffness, both patients were admitted to the hospital for observation and ultimately discharged home the following day.

*Population Health Research Capsule*

What do we already know about this clinical entity?

*Adenovirus is a common respiratory pathogen with varied clinical presentations and the potential to cause severe disease*.

What makes this presentation of disease reportable?

*This is the first known presentation of neuroinvasive adenovirus in otherwise healthy adults*.

What is the major learning point?

*There is the potential for severe complications in the presence of adenovirus infections*.

How might this improve emergency medicine practice?

*Emergency physicians should be aware of emerging pathogens and new presentations of known respiratory infections*.

Patient five was a 20-year-old male who presented to the ED for extremity weakness and cough. He was a close contact of three of the previous patients and presented on the advice of family and friends who had knowledge of the disease severity in the prior cases. A physical exam did not reveal any meningismus or focal neurologic abnormalities, and vital signs were within normal limits. His exam was notable for tenderness in the lower extremity muscle groups. His respiratory PCR testing was notable for adenovirus infection. There was concern for viral myositis vs rhabdomyolysis, confounded by a history of recent strenuous exercise. A CK was elevated to greater than 7000 international units per liter; however, renal function and electrolyte testing were normal. The patient was admitted and discharged two days later. The patient did not develop any signs of meningoencephalitis during admission, and a LP was not performed.

Patient six was a 20-year-old male with a PMH of Crohn’s disease on immunosuppressive therapy who presented shortly after patient five with symptoms of fever, cough, myalgias, and malaise. He was a close contact of several of the prior patients and was aware of the severity of their illnesses. Respiratory PCR testing was positive for adenovirus. Physical exam and vital signs were within normal limits. Lab evaluation was without notable findings; given the absence of CNS symptoms a LP was not performed. The patient was discharged with close outpatient follow-up and did not require admission.

Given the similarity and severity of the above presentations, campus health officials were alerted, and close contacts were notified. Expanded testing ultimately revealed over 80 individuals with confirmed adenovirus infection by respiratory PCR testing. Many additional individuals reported similar symptoms but did not undergo testing. No additional hospitalizations or ED evaluations were reported.

In summary, six patients were evaluated in the ED over three days. Lumbar puncture was performed in four of six cases with a finding of lymphocytic pleocytosis. Adenovirus serum and CSF testing was negative in all patients with suspected neuroinvasive disease. Two critically ill patients received cidofovir. While all patients largely recovered from their illness, patient one had lingering difficulties with executive function and memory.

## DISCUSSION

Neuroinvasive adenovirus infections are extraordinarily unusual in healthy patients. Adenovirus is a non-enveloped deoxyribonucleic acid (DNA) virus that is typically spread by inhalation of aerosolized droplets, but fecal-oral spread or contact with exposed surfaces can also produce infection. As the virus is non-enveloped, it is resistant to many disinfectants, although alcohol-based products remain effective.^1^ Outbreaks are typically seasonal, often occurring in the late fall or early spring. Typical symptoms include fever, pharyngitis, and cough with conjunctivitis and gastrointestinal (GI) symptoms being less common. In immunocompromised patients, adenovirus can cause more severe manifestations including pneumonia, hemorrhagic cystitis, nephritis, and meningoencephalitis.^2^ Adenovirus infection is a common concern in recipients of both solid organ and hematopoietic stem cell transplants. Depletion of T cells in preparation for transplant is a risk factor for severe infection. Congenital immunodeficiencies are also associated with severe adenovirus infection.

Meningoencephalitis is one of the most uncommon presentations of adenovirus. A single-center review reported meningoencephalitis symptoms in approximately 1% of all adenovirus cases, noting the majority of cases were seen in children age five or less.^3^ In cases of presumed viral encephalitis, evaluation for adenovirus is generally only considered once herpes simplex virus, varicella zoster virus, arboviruses, and other common causes of aseptic meningitis have been ruled out.^4^ Neurologic manifestations of adenovirus infection have been identified, but nearly all cases occurred in pediatric populations or those with predisposing conditions. A review of cases over 21 years identified 48 cases of adenovirus-associated CNS disease in immunocompetent children; the most common manifestations were febrile seizures, encephalitis, aseptic meningitis, and acute disseminated encephalomyelitis. Interestingly, like the patients in this case, 85% had virus detected in the respiratory or GI tract but not in the CSF.^5^

In a three-year review of adults and children presenting with meningoencephalitis, four adults and one newborn were found to have CSF positive for adenovirus; of the adults, two had HIV, one had sickle cell disease, and one had poorly controlled diabetes.^6^ A case report in 2006 identified an immunocompetent patient with a history of medulloblastoma status post resection and recent brain irradiation who presented with confusion, difficulty speaking, and increasing somnolence. After the patient developed seizures, a brain biopsy was obtained that was positive for adenovirus. The authors noted that this was the first case of adenovirus encephalitis without prior respiratory involvement. Lung biopsies failed to grow the virus, and the pathology was not typical.^7^ The isolated encephalitis seen in our two previously healthy young adults is unprecedented.

Treatment decisions in these cases were made more challenging by the fact that there are currently no approved treatments for adenovirus; no randomized controlled trials comparing therapies exist, and most of the literature focuses on pediatric populations or the highly immunocompromised. Ribavirin and ganciclovir have in vitro activity against adenovirus but have not been shown to be effective clinically.^2^ The most common agent is cidofovir, a cytosine nucleotide analogue that inhibits the viral DNA polymerase, but this treatment is generally reserved for cases of adenovirus in solid organ or hematopoietic transplant patients.^2^ There is no clear consensus on when treatment is appropriate, as spontaneous viral clearance even in the immunocompromised is not uncommon. Treatment is typically initiated for those with severe disease, disseminated infection, or profound immune deficits.^2^

In the two severe neuroinvasive cases presented here, the decision to treat was based on the neurologic impairment seen on admission. As nothing is known about the natural history of neuroinvasive adenovirus in healthy individuals, it is unclear how much impact the cidofovir had on their clinical improvement.

## CONCLUSION

Adenovirus is a common pathogen in our society often associated with mild respiratory illness. In this case series, we report the development of significant encephalitis from a neuroinvasive strain of adenovirus within a college community. As respiratory viruses continue to challenge our healthcare systems, new spectrums of disease are being discovered. We believe clinicians should be aware of the potential for serious neuroinvasive adenovirus disease in their evaluation and management of patients presenting with respiratory and central nervous system symptoms. This series also shows the role of the ED in population health surveillance. Early detection and reporting of these atypical presentations aided public health officials in rapidly expanding surveillance systems and contact tracing. This further emphasizes the multidisciplinary and ever evolving role of the ED within the larger healthcare system.
